# The Role of PARP1 in Monocyte and Macrophage Commitment and Specification: Future Perspectives and Limitations for the Treatment of Monocyte and Macrophage Relevant Diseases with PARP Inhibitors

**DOI:** 10.3390/cells9092040

**Published:** 2020-09-06

**Authors:** Maciej Sobczak, Marharyta Zyma, Agnieszka Robaszkiewicz

**Affiliations:** 1 Department of General Biophysics, Faculty of Biology and Environmental Protection, University of Lodz, Pomorska 141/143, 90-236 Lodz, Poland; maciej.sobczak@unilodz.eu; 2 Department of Immunopathology, Medical University of Lodz, 7/9 Zeligowskiego, Bldg 2, Rm177, 90-752 Lodz, Poland; marharyta.zyma@stud.umed.lodz.pl

**Keywords:** poly-ADP-ribose polymerase 1 (PARP1), macrophages, polarization, differentiation, stem cells

## Abstract

Modulation of PARP1 expression, changes in its enzymatic activity, post-translational modifications, and inflammasome-dependent cleavage play an important role in the development of monocytes and numerous subtypes of highly specialized macrophages. Transcription of *PARP1* is governed by the proliferation status of cells at each step of their development. Higher abundance of PARP1 in embryonic stem cells and in hematopoietic precursors supports their self-renewal and pluri-/multipotency, whereas a low level of the enzyme in monocytes determines the pattern of surface receptors and signal transducers that are functionally linked to the NFκB pathway. In macrophages, the involvement of PARP1 in regulation of transcription, signaling, inflammasome activity, metabolism, and redox balance supports macrophage polarization towards the pro-inflammatory phenotype (M1), which drives host defense against pathogens. On the other hand, it seems to limit the development of a variety of subsets of anti-inflammatory myeloid effectors (M2), which help to remove tissue debris and achieve healing. PARP inhibitors, which prevent protein ADP-ribosylation, and PARP1‒DNA traps, which capture the enzyme on chromatin, may allow us to modulate immune responses and the development of particular cell types. They can be also effective in the treatment of monocytic leukemia and other cancers by reverting the anti- to the proinflammatory phenotype in tumor-associated macrophages.

## 1. Introduction

The differentiation of stem and intermediate tissue-specific precursors into more specialized cell types requires quantitative and qualitative adaptation of the cellular proteome. The desired profile of proteins capable of driving cell conversion and gaining the final phenotype is acquired by the shift to alternative transcriptional profiles, but also post-transcriptional mechanisms. Since cell-type-specific genes inactive in precursors require activation upon differentiation and stemness-related transcription factors must be silenced, the proper composition of RNAs for further processing is ensured by the plasticity of transcriptional profiles. The chromatin responsiveness to stimuli that induce cell specification is mainly controlled by epigenetic regulation, which includes histone and DNA modification, and by the set of developmental transcription factors. The co-regulatory role in transcription and in post-transcription control of gene expression was assigned to poly(ADP-ribose) polymerase 1 (PARP1) over two decades ago, when over 30 nuclear proteins such as histones, RNA polymerases 1 and 2, hnRNP, topoisomerases, HMG proteins, and some transcription factors were identified as acceptors of ADP-ribose polymers as reviewed by D’Amours et al. [1]. The discovery of three zinc fingers in the N-terminal part of the protein that is known as the DNA-binding domain confirmed that the enzyme can interact with DNA and act directly on the chromatin [2]. PARP1 belongs to the family of poly(ADP-ribose) polymerase. Most of the family members that are reviewed in Jubin et al. are capable of transferring a single unit of ADP-ribose or multiple ADP-ribose moieties from NAD+ to proteins [3]. This results in protein MARylation or PARylation, respectively. PARP1 falls in the latter category, but its regulatory role goes beyond enzymatic activity and also comprises physical interaction with numerous proteins. A large number of reports have described the PARP1 contribution to DNA repair, and the enzyme is most frequently referred to in this context. However, numerous papers have provided evidence on the role of PARP1 in a wide range of intracellular processes, including transcription. The hypothesis of PARP1 involvement in inflammatory responses came about via detailed descriptions of the enzyme’s role in transcription of pro-inflammatory factors in primary macrophages and microglia [4,5,6]. Later reports documented the fluctuation of PARP1 expression during the differentiation of embryonic, hematopoietic stem and progenitor cells to monocytes and macrophages and the involvement of the enzyme in defining the phenotype of particular cell types in the hematopoietic lineage development [7,8,9,10]. The understanding of PARP1′s role in the differentiation pathway may allow us to intentionally modulate the commitment of hematopoietic precursors, interfere with the development of monocytes and macrophages, and disrupt macrophage polarization. Currently available PARP inhibitors are capable of preventing ADP-ribosylation of the target proteins and capturing the enzyme on the chromatin. This gives a relatively wide range of possibilities to interfere with monocyte and macrophage specialization. However, the lack of inhibitor specificity resulting from the relatively high homology between PARP1, PARP2, and PARP3 underlies the need for further discrimination between the role of PARP1 and other family members in the intracellular processes that characterize hematopoietic precursors and myeloid effector cells.

## 2. The Origin of Monocytes and Macrophages

Embryonic stem cells (ESC) with unrestricted differentiative potential develop into more specialized cells such as hematopoietic stem/progenitor cells (HSPC), which in turn give rise to all blood cell lineages. HSC precursors proliferate rapidly in order to prepare for relocation to the liver. According to the reviews by Domaratskaya et al., Ciriza et al., and Ng et al., mature HSCs migrate from the liver to the bone marrow, where they differentiate into any type of blood cell: those involved in oxygen delivery, e.g., red blood cells; those maintaining hemostasis, e.g., platelets; and immune cells that protect the host from pathogens [11,12,13]. Granulocyte and macrophage progenitors tend to transform into precursors of monocytes under the influence of M-CSF (CSF1) cytokine. M-CSF stimulates the activity of PU.1 transcription factor, which directs HSC on differentiation into myeloid cells. Besides PU.1, there are several other proteins involved in the HSC-monocyte transition, e.g., KLF4 and nuclear receptor NR4A1/Nur77, which was reviewed by Swirski et al. [14]. Additionally, Pittet and co-authors, in their review, stated that CX3CR1 and CCR2 receptors that are expressed on the surface of monocytes as well as on their precursors may also be involved in their differentiation [15]. Later differentiation of monocytes into anti-inflammatory macrophages (M2) is controlled by M-CSF, as reviewed by Italiani et al. [16]. The process described above occurs in a physiological state, in which HSCs differentiate in a balanced fashion into any of the blood cells. However, that can be altered during ongoing inflammation. In a review by Pietras and co-authors, they claim that, through inflammatory signals including toll-like receptor (TLR) signaling, interleukin-1 (IL-1), interferons (IFNs), and tumor necrosis factor (TNF), the balance is tilted towards differentiation into platelets or myeloid cells [17]. In the early stage of inflammation, monocytes recruited to the site of inflammation are more likely to develop into proinflammatory macrophages (M1) than M2, which is orchestrated by GM-CSF (CSF2), in order to fight ongoing infection. Conversely, monocytes recruited to the inflammation site in the later stages of inflammation tend to differentiate into M2 macrophages to quench the inflammatory process as reviewed by Italiani et al. and Yang et al. [16,18].

M2 macrophages can be divided into three major subpopulations: M2a, M2b, and M2c. M0 macrophages stimulated by IL-4 and/or IL13 polarize into the M2a subtype, which is characterized by the expression of, inter alia, arginase-1, IL-4Ra, TGFβ, and CD206. The M2b phenotype, which actively transcribes IL-10, TNFα, IL6, and IL1β, can be activated by immunocomplexes in combination with or without LPS. The last phenotype, M2c, can be achieved by macrophage stimulation with IL10 and glucocorticoids, which leads to the expression of IL10, TGFβ, and VEGF [19]. One more M2 subtype that is known as a tumor-associated macrophage is classified as M2d. It develops in response to tumor-derived factors and releases IL10, IL12, TNFα, TNFβ, VEGF, CCL20, CCL22, and MMP9, thereby supporting tumor growth and metastases and acting as an immunosuppressant.

Interestingly, some macrophages develop in ways other than the above described pathway and differentiate directly from stem cells during the fetal period. Regarding the origin, two populations of macrophages can be distinguished: monocyte-derived macrophages, the differentiation of which is described above, and tissue macrophages, which are derived directly from myeloid precursors that originate from the yolk sac and fetal liver. Notably, Gordon and co-authors, in their review, state that if the tissue macrophage population is depleted, monocytes can differentiate into their successors [20].

The path of monocyte and macrophage differentiation is accompanied by the loss and gain of some cellular features including pluripotency, specification, and functions in the immune system. All these attributes are, to a certain extent, controlled by PARP1. These include the self-renewal of precursors, activity of transcription factors, pattern of surface markers, and downstream signaling cascades in monocytes and macrophages. Activation of TLRs or other cytokine and chemokine receptors leads to the synthesis and release of immunomodulatory mediators and pathogen-killing agents such as reactive oxygen species.

## 3. PARP1 Transcription Reflects Changes in Proliferation Status and Requirement for DNA Protection in Monocytes, Macrophages, and Their Precursors

### 3.1. PARP1 Transcription Is Driven by the Promoter Characterized by the E2F Binding Motif, Which Links Gene Transcription with Mitotic Divisions

**Figure 1:**
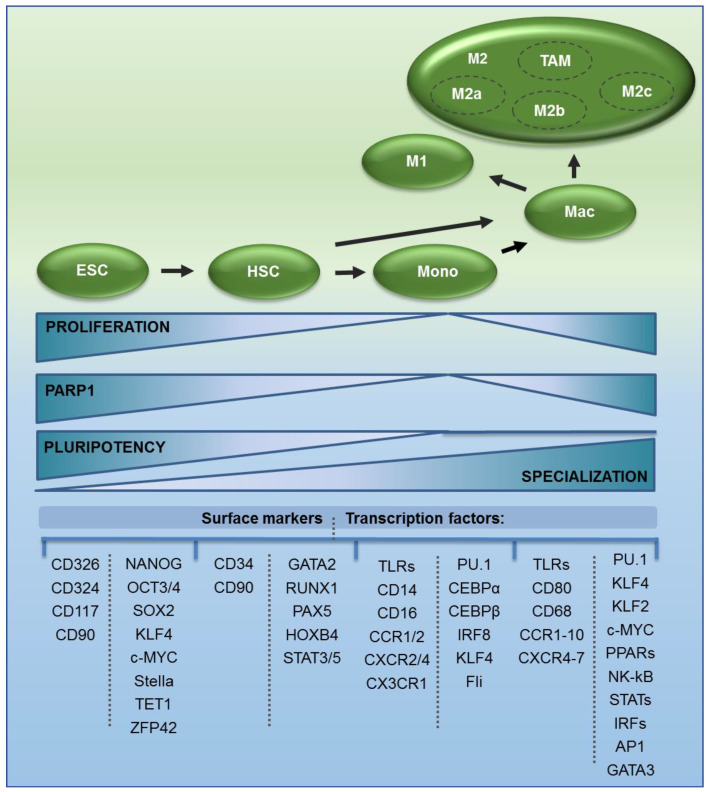
The interdependence between proliferation, specialization, and PARP1 expression during monocyte and macrophage development. The specialization of myeloid effector cells like monocytes and macrophages is associated with a gradual loss of pluripotency and self-renewal. The latter ability is restored in some macrophages that are capable of self-replenishing. The proliferation potential of particular cell types reflects PARP1 abundance since the *PARP1* transcription is controlled by cell transition from G1 to S phase during the cell cycle progression. Monocyte and macrophage development is followed by transcriptional reprogramming, which involves the various sets of transcription factors and modification of the pattern of surface markers.

Transcription of *PARP1*, as with many other genes involved in DNA repair, is controlled by cell cycle progression. The *PARP1* promoter is characterized by the presence of a CpG island and the binding motif for the E2F family of transcription factors, which can replace each other and define the composition of repressive complexes, depending on the mode of cell cycle arrest. The monocyte differentiation model, as well as myoblasts and cancer cell lines, reveals a high abundance of the enzyme in quickly proliferating cells and a low abundance upon exit to G0 and G1 but not G2 arrested cells (Figure 1) [21,22,23].

Although data on PARP1 transcription in dividing embryonic stem cells versus other myeloid progenitors or specialized cells are missing, the role of the PAR-synthesizing enzyme in maintaining their stemness was confirmed and will be described in the following section [24]. The results of the abovementioned study indicate that the abundance of the enzyme in embryonic stem cells must be substantial to make this enzyme critical for safeguarding pluripotency by occupying key pluripotency genes. Furthermore, the transcription of *PARP1* decreased gradually over eight days of differentiation induced by LIF withdrawal and culture on nonadherent plates. The PARP1 level was visibly higher in embryonic stem cells than in lineage-committed trophoblast stem cell lines. PARP1 deficiency favors the development of all three germ layers, as well as the mesoderm, which gives rise to hematopoietic stem cells. This suggests that PARP1 abundance in proliferating HSC might be an intermediate between ES, which are capable of unlimited growth and fast self-renewal, and growth-arrested monocytes. In culture, human CD34+ myeloid progenitors stimulated with a mixture of SCF, IL3, and IL6 proliferate and express the enzyme to a higher extent than blood-derived monocytes [23].

The differentiation to monocytes dramatically reduces the expression of factors that determine the pluri/multipotency and self-renewal potential of progenitor cells (remaining levels of embryonic OCT4, SOX2, NANOG, ZFP42, and hematopoietic GATA2, RUNX1, and PAX5), but is followed by overexpression of shared (PU.1 and CEBP/α) and specific lineage determinants (IRF8, KLF4, Fli1, and C/EBPb), as reviewed by Zhu et al. [25]. C/EBPα transactivates lineage-specific differentiation genes and inhibits monocyte proliferation in G0/G1 by repressing E2F-regulated genes [26]. These are represented by cyclins, cyclin-dependent kinases, PCNA, and many others that promote cell transition to the following cell cycle phases. Inhibition of HSC divisions in G1 leads to considerable PARP1 repression, the same as their differentiation to monocytes. In spite of the same outcome (cell cycle arrest before S phase), the molecular mode of gene suppression varies. G1 arrest induces the enrichment of E2F1 at the gene promoter and the recruitment of RB1, HDAC1, PRC2, and BRM/BRG1-based SWI/SNF. It results in histone deacetylation and the trimethylation of H3K27—both capable of gene repression. In G0-arrested monocytes, replacement of E2F1 with E2F4 is followed by the binding of RBL2, HDAC1, and BRM-based SWI/SNF. In both cases, inhibitors of histone-remodeling enzymes increase PARP1 transcription, suggesting that the observed modifications caused by the growth inhibition can be transient and reverted.

PARP1 suppression in monocytes might also be linked to increased expression of PU.1, which binds to the basal promoter of poly(ADP-ribose) glycohydrolase (PARG), downregulates promoter activity, and transiently declines gene transcription [27]. The PAR-decomposing enzyme was found to be crucial for the promoter activity of the *PARP1* gene [28]. Therefore, PU.1 may contribute indirectly to *PARP1* silencing by repressing PARG, but such a hypothesis requires experimental verification. PU.1, by itself, was not considerably enriched at the *PARP1* promoter in the model of PMA-induced monocyte/macrophage differentiation [23].

The hypothesis of the reversible and transient *PARP1* repression in monocytes finds confirmation during monocyte differentiation to macrophages. The latter cells enter mitosis in response to M-CSF as well as GM-CSF stimulation of their progenitors and in mature form express markers such as Ki67, PCNA, cyclin B, and cyclin E, which are hallmarks of mitotic cells [10,29]. M-CSF activates a self-renewal gene network comprising *Klf2*, *Klf4*, and *Myc*, which are controlled from PU.1-dependent cell-type-specific enhancers [30]. Proliferation is also induced by oxLDL, phagocytosis, efferocytosis, neuropeptide FF, interleukin 1, and *Cryptococcus*. The re-entrance of the cell cycle restores the transcription of *PARP1* and other genes involved in the removal of DNA lesions and brakes. A study on their regulation revealed that the composition of their promoters changes during monocyte-to-macrophage differentiation. HDAC1, which persists at the gene promoters in both cell types, is accompanied by EP300 and BRG1. The proliferation rate dictates the activity of particular histone acetylation-defining components: divisions inhibit HDAC1, stimulate EP300, and increase nucleosome acetylation and their removal by BRG1, thereby promoting transcription, whereas G0 and G1 arrest shifts the histone status towards deacetylation, chromatin compaction, and gene suppression. Such a fine-tuning of histone acetylation, but not protein composition, allows the cell to quickly respond to alterations in the proliferation rate and to differentiation. In dividing macrophages, the described mechanism renders these cells resistant to the accumulation of DNA damage.

Further macrophage polarization to the pro-inflammatory phenotype by LPS, IFNγ, or *Mycobacterium* results in immediate growth arrest due to the repression of cell cycle “checkpoint” genes, which eventually block G1/S transition. The proliferation status of M2 macrophages seems to depend on the subtype and/or polarization-inducing factors, since even one M2-polarizing cytokine IL4 is reported to both stimulate and inhibit proliferation. During macrophage activation with LPS and their polarization to pro-inflammatory phenotype, *PARP1* transcription is again positively correlated with proliferation status, since LPS-induced growth arrest is followed by a decline in PARP1 abundance. Although the mechanism in this particular case has not been studied yet, it is possible that the EP300-HDAC1-BRG1 and E2F-RB regulatory circuits described above also operate this type of cell terminal specialization. Nothing is known about the PARP1 level in M2 macrophages, although according to data from the BLUEPRINT project the gene expression is comparable across all three types of macrophage (M0, M1, and M2). This further suggests that *PARP1* repression depends on the stimuli and/or macrophage origin (monocyte-derived vs. tissue-specific) rather than on the profile of macrophage polarization.

Importantly, PARP1 abundance in cells is not solely controlled by its transcription, but also by other post-transcriptional processes such as mRNA targeting by miRNA, protein stability, and degradation that should be also taken into consideration [31]. However, data on *PARP1* mRNA and protein turnover during monocyte and macrophage differentiation are missing.

### 3.2. PARP1 Regulatory Proteins Define the Contribution of the Enzyme to DNA Repair and Transcription by Discriminating Amino Acid Residues for Poly(ADP-ribosyl)ation

The primary role of PARP1 in stem and other cell types is assigned to genome protection, whereas transcription control is considered secondary to the first aspect. ESC and multipotent adult stem cells (ASC) have stringent control of their genome integrity to avoid the propagation of unpaired lesions during their mitotic divisions. According to the reviews by Fortini et al. and Nouspikel et al., as well as a research study conducted by Wiśnik et al., there are more efficient DNA repair systems in proliferating stem and progenitor cells, and the differentiation-associated loss of cell capability to cope with DNA lesions has been experimentally confirmed [23,32,33]. PARP1 and some other proteins involved in DNA repair are transcriptionally controlled by the cell proliferation status. Therefore, stem cells that undergo mitotic divisions are characterized by a relatively high abundance of these proteins. Once the cell gains the desired phenotype and accomplishes differentiation, the rigorous control of DNA brakes and removal of mismatched nucleotides or their covalent modifications become less essential and, with very few exceptions, the expression of genes responsible for genome protection undergoes suppression in a growth arrest-dependent fashion. This perfectly reflects the situation of PARP1 and other proteins with a similar function in DNA damage showing a decreased response during monocyte development. Elevated ROS production in phagocyting macrophages induces internal pressure to protect the genome and pass through all checkpoints to accomplish mitotic divisions. The self-replenishing potential of phagocytes is responsible for the reinstatement of efficient genome defense by increasing the transcription of *PARP1* and other genes involved in DNA repair. This suggests that the modulation of PARP1 expression during monocyte and macrophage development has a multifaceted meaning, predisposing each cell type in the differentiation path to its physiological function.

The co-regulatory role of PARP1 in transcription control has been widely described in reviews by Kraus and Kraus et al., and comprises a variety of aspects, starting with direct interaction with transcription factors, chromatin remodeling enzymes, co-factors of transcription machinery, their ADP-ribosylation, and modifications of histones, which affect chromatin compaction and transcription efficiency [34,35]. Although Lys, Arg, Glu, Asp, Cys, Ser, Thr, Ser, His, and Tyr residues were proposed as ADP-ribose acceptor sites by proteomic approaches, as reviewed by Alemasova and Lavrik, the mechanism that dictates site-specific PARylation remained elusive until recently [36]. ADP-ribosylation of particular amino acid residues upon DNA damage and transcriptional activation depends on the regulatory proteins and the interaction of particular domains of PARP1 with DNA and nucleosomes. A study of the multiple deletional isoforms of PARP-1 revealed a short-term binding of the DNA-binding domain and activation of PARP-1 by the damaged DNA [37]. The synthesis of poly-ADP-ribose by PARP1 in response to DNA lesions requires HPF1, which is specific to the DNA damage response and switches the amino acid specificity of PARP1 and PARP2 from aspartate or glutamate to serine residues [38,39,40]. The specific localization of PARP1 around transcription start and termination side is governed by the interaction between the C-terminal domain of PARP1 and histone H4 in a DNA-independent fashion. Transcription-associated, long-term activation of PARP1 results in a continuous accumulation of pADPr and facilitates the transcriptionally permissive state of chromatin [37]. Neither activity nor the DNA-binding domain was necessary for the interaction of PARP1 with NFκB and the stimulation of gene expression in response to pro-inflammatory stimuli such as TNF or LPS [41]. As transcription activator PARP1 ADP-ribosylates EP300 and BRG1 in proliferating cells, it co-occurs with acetylated histones genome-wide [42]. However, upon DNA damage, acetylation is immediately removed by HDACs since this modification blocks the DNA damage-induced ADP-ribosylation of histone H3 serine 10 and repair of the lesions [43]. Similarly, ADP-ribosylation of serine interferes with the acetylation of adjacent lysine residues [44]. This indicates another method of cross-talk between DNA repair and transcription control by PARP1, which might be of particular importance in proliferating monocyte progenitors and macrophages for the reasons described above. Regardless of the acceptor residue, the accumulation of free poly-ADP-ribose polymers regulates inflammatory responses in macrophages and defines their polarization routes, as reviewed by Pazzaglia et al. [45].

## 4. PARP1 in Precursors of Monocytes and Macrophages—A Driver of Pluri- and Multipotency

In proliferating embryonic stem cells, the orchestrated action of two PARPs, PARP1 and PARP7, protects *Nanog*, *Oct4*, *Sox2*, *Stella*, *Tet1*, and *Zfp42* from progressive epigenetic repression. In detail, *Parp1 (-/-)* embryonic stem cells were characterized by a lower expression of Nanog, Oct4, Sox2, Tet1, Tet2, and Tbx3, as well as Stella in a Zfp41-high background, due to the increased accumulation of epigenetic repressive markers H3K9me3 and H3K27me3, and (to a lesser extent) by preventing DNA methylation at pluripotency factor loci [24]. The deficiency of the enzyme resulted in the increased propensity to differentiate into derivatives of all three germ layers during embryoid body formation, which was associated with elevated mRNA levels of *Foxa2*, *Gata2/4/6*, *Runx1*, *Sox1*, *Ascl1*, and *Zic1*. In addition to maintaining the expression of key pluripotency factors, PARP1 facilitates their recruitment to chromatin. As described in Liu and Kraus, the PAR-synthesizing enzyme co-localizes with SOX2 and OCT4 genome-wide, but physically interacts with only the first of the two listed transcription factors and increases the transcription of *NANOG*, *KLF4*, and *TBX3* [46]. *Parp1 (-/-)* was severely impaired in the expression of Nanog, but was characterized by an increased level of differentiation markers. PARP1′s effect on the expression of pluripotency genes did not involve ADP-ribosylation since PJ34 did not alter their transcription. Sox2 binding sites that require PARP1 (independent of its catalytic activity) for the binding of Sox2 have a specific set of features that include high nucleosome density, less optimal Sox, fewer composite Sox/Pou motifs, and co-binding by fewer additional transcription factors, namely intractable genomic loci. In another example, oligo(ADP-ribose) polymerase activity is required for PARP1-/KLF4-mediated induction of the *TERT* gene, the product of which contributes to the maintenance of self-renewal in ESCs by facilitating the expression of *NANOG* and repressing endoderm differentiation genes *Gata6* and *Sox17* [47].

*PARP1* germline deficiency promotes ES cell commitment to the mesoderm, and PARP1 expression negatively correlates with, inter alia, GATA2 and RUNX1, which characterize hematopoietic stem cells [24]. On the other hand, PARP-synthesizing enzyme is an activator of *GATA2* and *RUNX2* transcription, since the reinstatement of PARP1 transcription in human monocytes by targeting RBL2 or HDAC1 considerably increased the expression of these transcription factors [23]. This suggests that a decline in PARP1 abundance is necessary for the repression of pluripotency transcription factors, for embryonic stem cell commitment to particular germ layers, and for the induction of lineage-specific genes. The latter might be still under the transcriptional control of PARP1 in proliferating hematopoietic stem and precursor cells since, in monocytes, which are low in PARP1, the enzyme still positively regulates numerous genes that are crucial for the phenotype and function of myeloid effectors—surface receptors and signaling cascade mediators downstream of TLRs [48]. *PARP1* suppression, which follows hematopoietic stem cell differentiation to monocytes, is associated with the further repression of embryonic *OCT4*, *SOX2*, *NANOG*, *ZFP42*, and hematopoietic transcription factors *GATA2* and *RUNX1*. The observed increase in the expression of all enumerated transcription factors upon PARP1 reinstatement in monocytes supports the idea of a causative sequence of cell cycle-dependent declines in the expression of PARP1, embryonic and hematopoietic transcription factors, and monocyte development.

## 5. PARP1 in Monocytes—The Role of Enzyme Repression in Transcription Control of Inter- and Intracellular Signaling Mediators

**Figure 2:**
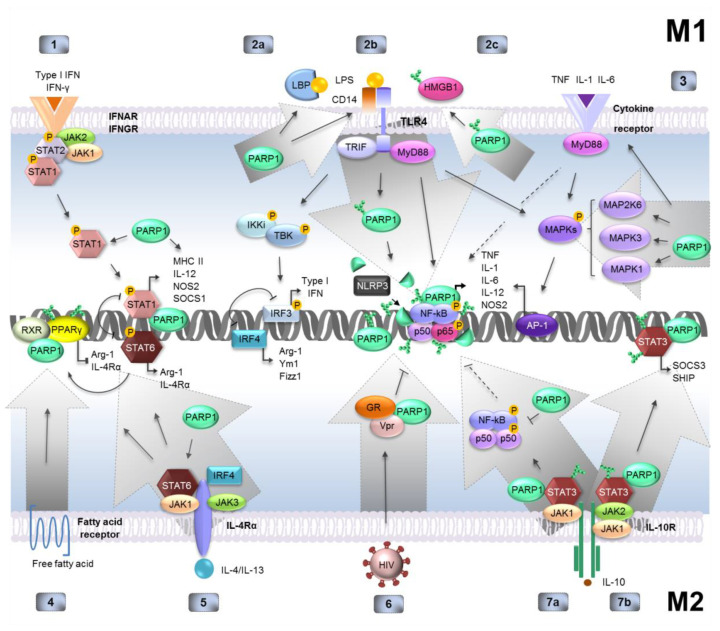
The contribution of PARP1 to key pathways associated with M1 and M2 polarization of macrophages. The signaling cascades induced by cell surface receptors end at the chromatin and result in the transcription of genes that are typical of the proinflammatory (M1) or anti-inflammatory (M2) phenotype. The physical interaction of PARP1 with other proteins is indicated by di- or multisubunit complexes of the enzyme. Their position in the scheme reflects the intracellular localization of their physical and functional interaction (on the chromatin or outside of the genome). Whenever PARP1 modulates the activity of transcription factor and the expression of target genes, the enzyme is presented with a transcription factor on chromatin. PARP1 appearing alone on the DNA strand is a hallmark of the modification of chromatin accessibility. ADP-ribosylation of proteins is depicted by chains of green balls. The unknown mechanism of functional impact of PARP1 on some proteins is indicated by arrows when PARP1 targets are positively controlled by the enzyme, and dashes depict an inhibitory effect of PARP1. A detailed description of cellular pathways (marked 1‒7) and the role of PARP1 is provided in the text.

The transcription of PARP1 and other genes that are involved in the repair of DNA damage, and are transcriptionally controlled by the status of proliferation, decline during monocyte development. As described in the previous paragraph, the decrease in PARP1 abundance supports the loss of pluri- and multipotency as well as self-renewal capacity, but also allows the transcription of genes that were repressed by this enzyme in monocyte progenitors. Although relatively low, the remaining level of PARP1 is still targetable by siRNA and PARP inhibitors. It positively regulates the expression of genes that are crucial for monocytes and possibly impacts intracellular processes by ADP-ribosylation. For example, three PARP inhibitors, AIQ, olaparib, and EB47, were shown to attenuate monocyte adhesion to the brain endothelium and to decrease their adhesion/migration across BBB models. These compounds prevented the conformational activation of VLA-4 and LFA-1 integrins and inhibited RhoA and Rac1 GTPases [49]. This suggests that protein ADP-ribosylation contributes to inter- and intracellular signaling routes, but the use of inhibitors that lack specificity towards PARP1 does not allow us to attribute the observed role of ADP-ribosylation only to PARP1 enzymatic activity. A recent study on human monocytes provided further evidence of the role of PARP1 and its differentiation-induced repression in the adaptation of these cells to receiving and transmitting the external stimuli to chromatin. Numerous genes encoding surface receptors and TLR downstream signaling mediators are, the same as *PARP1*, suppressed by RBL2-E2F4-HDAC1-BRM units in a cell-cycle-dependent fashion [48]. However, 24 of the 28 identified genes controlled by RBL2 repressive complexes are co-regulated by PARP1. In proliferating cells and upon RBL2 silencing, the enzyme enables the recruitment of EP300, which acetylates nucleosomes, to the gene promoters. This activates the expression of genes such as *MAP2K6* and *MAPK3* (Figure 2, regulatory scheme 3). Their products act upstream of p38 and ERK kinases—key components of the MAPK cascade. Thus, RBL2 defines some aspects of monocyte phenotype by repressing *PARP1* and, in this facet, the decline of ADP-ribosylase may provide an additional mechanism that protects monocytes from overactivation and from the excessive expression of pro-inflammatory cytokines [50]. PARP1 also acts as a positive regulator of other genes involved in canonical NFκB signaling such as *AKT1* and *MAPK1*, the product of which is known as ERK2, and *IKBKB*. The last gene encodes a protein that phosphorylates IKK, an inhibitor of p65/NFKB3-p50/NFKB1. The phosphomodification of IKK leads to its ubiquitination, proteasomal degradation, and to the release of the functional p65 and p50 dimers, which are capable of binding to gene regulatory elements and activating transcription, as reviewed by Schmid et al. [51]. In contrast, PARP1 represses *NFKB1*, which is transcribed and post-transcriptionally processed to give p50, homodimers of which inhibit the transcription of various genes activated by canonical NFκB (Figure 2, regulatory scheme 7a). The p50‒p50 homodimers interfere with the recruitment of p65/NFKB3-p50/NFKB transcription factors.

In addition to intracellular transmitters, PARP1 contributes to the transcription control of a relatively high number of surface receptors. They allow for (a) cell association with certain immune functions and recognition by other cell types, (b) identification of pathogen components and immediate response, and (c) sensing the environment and recruitment towards the inflammation site or target tissue. The first group of functionally linked membrane proteins is mainly represented by clusters of differentiation, also known as classification determinants (CDs). These molecules include receptors, adhesion molecules, membrane-bound enzymes, and glycans that play multiple roles in development, activation, and differentiation [52]. Monocytes express various antigens depending on their stage of development, such as CD4, CD11b, CD13, CD14, CD15, CD18, CD34, CD36, CD45, and CD64 as well as the human leukocyte antigen DR isotype (HLA-DR) [53]. CD14, which, together with LPS binding protein (LBP), helps to recognize bacterial glycans by Toll-like receptors, is generally approved as a monocyte marker. There are very few unique macrophage markers because of various macrophage subsets and the conditions of their local environment. M1 are often characterized by CD80 and CD68. The (b) group of surface receptors, mostly represented by Toll-like receptors responsible for pathogenic antigen recognition, appears on monocytes, M1 and M2c macrophages. The highest expression of TLR2 is a hallmark of monocytes. In addition to TLR2, they express TLR1, TLR4, TLR5, TLR6, and TLR8, and low but measurable levels of TLR9 [54]. The expression of particular TLRs differs between monocytes and macrophages. Group (c), which orchestrates the recruitment of cells towards inflamed tissue and coordinates the joint action of multiple immune cell types, is represented by receptors for immunomodulatory and extracellular signaling molecules such as chemokines and cytokines. Chemokine receptors are most frequently represented by C-X-C Motif Chemokine Receptors (CXCRs) and C-C chemokine receptors (CCRs). Classical monocytes CD14^+/^CD16^+^ express a variety of these on their surface, including the most commonly expressed, CCR1, CCR2, CXCR2, CXCR4, and CX3CR1, in contrast to the rarely expressed CCR5, CCR7, CCR9, CXCR1, and CXCR7. The lowest level of expression was observed in CCR3, CCR4, CCR6, CCR8, CCR10, CXCR3, CXCR5, CXCR6, XCR1, and ChemR23 [55]. Although both M1 and M2 macrophages express chemokine receptors, the mRNA levels of particular receptors differ between them. The mRNA levels of CCR2, CCR3, CCR4, CCR6, CCR8, CCR9, CCR10, CCR11, CXCR2, CXCR5, CXCR6, CXCR7, and CX3CR1 are superior in M1 compared to M2, while the opposite is the case for CCR1, CCR5, and CXCR4 receptors, whose mRNA levels are higher in M2 [56]. According to reviews by Hamilton and Lewko et al., cytokine receptors, depending on the type, stimulate pro- and anti-inflammatory responses and the development of M1 or M2 polarized cells (also from monocytes) [57,58]. The list of the most abundant cytokine receptors includes these for interleukins (ILRs), tumor necrosis factor receptor superfamily (TNFRSFs), and interferon-α/β as well as interferon-γ receptors (IFNARs and IFNGR, respectively).

For the great majority of them, PARP1 serves as a co-activator in monocytes. The list of genes downregulated upon silencing of the enzyme comprises Toll-like receptors (*TLR2* and *TLR3*, while *TLR9* is increased), *LBP*, receptors for interleukins, tumor necrosis factor, chemokines, nerve growth factor (*NGFR*), death receptor *FAS*, and monocyte markers *CD80* and *CD14* (Figure 2, regulatory scheme 2a). As described in the review by Sampath and co-authors, the pattern of surface receptors and their response to stimuli differ between classical (CD14++, CD16−), intermediate (CD14+, CD16+), and nonclassical (CD14+, CD16++) monocytes [59]. Thus, the role of PARP1 in defining the phenotype and function as well as *PARP1* transcription may vary between monocyte subsets, and further studies are required to describe their possible differences. PARP1 controls the transcription of a subset of genes that are crucial for the function of monocytes.

## 6. PARP1 in Macrophages—Its Role in Their Polarization

### 6.1. Factors That Activate PARP1 in Macrophages and Their Role in Macrophage Polarization

PARP1′s contribution to macrophage function and polarization comprises both physical interaction with other proteins and DNA as well as ADP-ribosylation of the targets. The shift to pro-oxidative conditions and increased ROS production in response to the invasion of pathogens challenges DNA, damage to which is among the top causes of PARP1 activation. As described in Section 3.2, DNA lesions due to the induction of specifically serine ADP-ribosylation may alter the profile of gene transcription and, thus, the macrophage proteome when compared to signaling and transcription-associated PARP1 activation [37,38,39,40]. The catalytic activity of the enzyme is also triggered in response to signals independently of DNA injury by post-translational protein modifications, which include acetylation, methylation, ubiquitination, sumoylation, and phosphorylation. Various macrophage subtypes are characterized by the presence of estrogen and progesterone receptor as well as hepatocyte growth factor (HGF), which is known as cMet [60]. Cell stimulation with progestin, a synthetic form of progesterone, leads to phosphorylation and thus activation of PARP1 by CDK2, which is active in the G1 phase of proliferating cells. A progesterone receptor lowers inflammatory responses by interfering with the NF-kB pathway, which is triggered by M1 polarizing agents (LPS, TNFα) and may, therefore, prevent the gaining of a pro-inflammatory phenotype and the accompanying inhibition of macrophage potential to self-renewal, as reviewed by Hall et al. [61]. Furthermore, estradiol, the ligand of ERα, induces PARP enzymatic activity and poly(ADP-ribosyl)ation of ERα. The covalent modification enhances the binding of the receptor to the estrogen response element that is present in the target gene promoter, thereby promoting ERα-mediated gene transcription. The impact of estradiol receptors on macrophage polarization seems to be debatable since contradictory results published in various papers show both attenuated production of pro-inflammatory cytokines including IL-6, TNFα, IL8, and macrophage inhibitory factor as well as upregulated expressions of the M1-associated proinflammatory factor inducible NO synthase (iNOS) and repressed expression of the M2-associated markers IL-10 and arginase in NR8383 macrophages [62,63]. PARP1 also undergoes activation upon stimulation of the cMet receptor for HGF. In Raw 264.7 macrophages, cMet receptor decreased the RNA level of LPS-induced *TNF-α*, *IL-1β*, and *iNOS* and enhanced that of *IL-10*. Hence, signaling from the cMet receptor is presumed to regulate macrophage transition to the M2 phenotype [64]. cMet, which belongs to the receptor tyrosine kinases, associates with and phosphorylates PARP1. Furthermore, Piao et al., in their review, claim that non-receptor tyrosine kinase Txk forms a trimolecular complex with PARP1 and EF1α, and directly phosphorylates the two co-occurring subunits. Such a complex has been found to specifically bind to the promoter of IFN-γ in vitro and provoke the gene transcription [65]. In macrophages, chromatin-bound PARP1 can be activated dually by intracellular signaling cascades that are triggered upon the binding of PAMPs, pro- or anti-inflammatory ligands, and by the mild oxidative environment that accompanies macrophage polarization [66]. The LPS-TLR2/4-MEK1/2-ERK1/2 signaling pathway leads to phosphorylation and activation of PARP1. LPS and type I IFN induced double-strand brakes and DNA damage response via ATM cascade. This most likely involves PARP1 activation, which in this case may be triggered dually by DNA brakes and signaling pathways, which lead to the activation of pro-inflammatory genes [67]. However, ERK also modifies key proteins involved in inflammasome function and leads to priming that precedes the activation of the NLRP3 inflammasome, which utilizes the activity of caspase-1 to cleave pro-IL1 and pro-IL18 and to generate their mature form that can be released from cells. NLRP3 and NLRC4 inflammasomes process full-length PARP1 into a fragment of 89 kDa in a stimulus-dependent manner in macrophages. PARP1 cleavage is catalyzed by inflammasome-associated caspases 1 or by the downstream inflammasome effector caspase-7 at the gene promoters, where PARP1 splitting facilitates the transcription of the subset of NFκB-dependent pro-inflammatory factors [68,69].

### 6.2. The Contribution of PARP1 to Classical Macrophage Polarization

The LPS/TNF-MEK-ERK-PARP1-RelA signaling pathway is responsible for the transcription of pro-inflammatory cytokines such as pro-interleukins IL-1β and IL-18, which are processed by the ERK-activated NLRP3 inflammasome prior to secretion to the extracellular space (Figure 2, regulator schemes 2b) [70,71]. In the described mode of the pro-inflammatory cascade, poly-ADP-ribosylation was induced canonically by ROS-DNA damage signal (LPS) and by the ERK pathway (LPS and TNF), and PARP1′s role in PAR synthesis was confirmed by its transient silencing with siRNA. PARP1 controlled expression of NK cell attractant CCL2 chemokine in macrophages upon vaccinia virus infection by affecting NFκB [72]. Induction of PARP1 enzymatic activity and modification of RelA/p65 enhances gene transcription at least fourfold: (a) ADP-ribosylation of NF-κB promotes its nuclear translocation and binding to its response sequences in macrophages; (b) depletion of NAD+ promotes NF-κB transcriptional activity by preventing RELA/p65 de-acetylation by another NAD+ consumer, SIRT1 deacetylase; (c) PARP-1 acetylation by p300/CBP enhances the functional interaction of RelA/p65-p50 with P300 and the mediator complex; thus, PARP1 can act as a promoter-specific coactivator of NF-κB; (d) histone ADP-ribosylation at the gene promoter disrupts the nucleosome structure, increasing the accessibility of DNA and recruitment of the transcription factor NF-κB [5,6]. This all results in robust transcription of the inflammatory cytokines [73]. A recent study on the identification of genome-wide ADP-ribosylation loci indicates that this modification is enriched in compacted chromatin, which is characterized by high nucleosome density and repressive modification of H3K9me3, but negatively correlates with histone modifications that are hallmarks of a transcriptionally permissive environment—H3K27ac, H3K9ac, and H3K4me1 upon oxidative stress [74]. These findings support the idea that ADP-ribosylated nucleosomes are evicted from transcriptionally active chromatin, whereas PARP1 preferentially binds to these regions [42]. Furthermore, this observation agrees with the previously described role of DNA damage-induced ADP-ribosylation of histones in transcription regulation and the establishing of the histone code [43,44]. Furthermore, PARP1 occurs in genomic regions characterized by H3K27ac(-)/H3K4me3(+) and H3K27ac(+)/H3K4me3(+), which correspond to poised and active enhancers, respectively. These regulatory regions, relatively distant from proximal gene promoters, play an essential role in macrophage development and function. Activation of macrophage-specific enhancers, and therefore the profile of gene expression, is regulated by the orchestrated action of lineage-determining transcription factors (LDTFs) and signal-dependent transcription factors (SDTFs). The latter are specified by the developmental origin of macrophages and tissue-specific signals. As a consequence, macrophages adopt a distinct phenotype by adapting transcriptional programs controlled by enhancer‒promoter interaction. PU.1 and C/EBP pretend to be major LDTFs during the development of macrophage and pioneering transcription factors due to the ability to prime inactive enhancers (induce monomethylation of H3K4) and improve the accessibility of these regions to signal-dependent TFs, as reviewed by Glass [75]. SDTFs, which activate the primed enhancers and gene promoters, increase nucleosome acetylation in a signal-dependent manner. They are represented by NFκB (RelA/p65-NFκB1/p50), STAT1, IRF3 and IRF5, and HIF1α in macrophages gaining the pro-inflammatory phenotype, whereas NFκB (NFκB1/p50-NFκB1/p50), STAT3, STAT6, IRF4, and HIF2α promote the anti-inflammatory phenotype. A recent paper documented the role of PU.1 and the distal enhancer in transcription control of *IL1β* in LSP-stimulated macrophages [76]. In response to TLR4 stimulation with bacterial endotoxin, the recruitment of NF-κB and further H3K27 acetylation of the gene promoter and the identified enhancer (located ~10 kbp upstream of the *IL-1β* transcription start site) was followed by PU.1-dependent enhancer-promoter looping and eRNA transcription from the active enhancer, which led to induction of cytokine transcription. Bearing in mind that NFκB-dependent transcription of *IL1β* is co-regulated by PARP1, PAR-synthesizing enzyme seems to be involved in promoter‒enhancer functional interaction. Advances in the area of enhancer impact on gene expression revealed increased enhancer‒promoter separation upon activation that, interestingly, was driven by poly(ADP-ribose) polymerase 1 in a model of embryonic stem cell differentiation to neural progenitor cells [77]. In light of all these reports, which indicate the role of PARP1 and ADP-ribosylation in pro-inflammatory responses of macrophages, the observed PARP1 repression during monocyte-derived macrophages activation with LPS is quite surprising [8]. However, pro-inflammatory cytokines belong to fast and moderately responding genes and canonical NFκB binds to their promoters within minutes to hours, whereas the substantial decline in PARP1 abundance as a consequence of gene repression is usually observed at later time points.

Some of the canonical NFκB target genes such as *GM-CSF*, *IL-6*, and *LIF* (interleukin 6 family cytokine) are repressed by chromatin-bound PARP1 in macrophages [78]. Their LPS-induced expression requires PARP1 cleavage at their promoters by caspase 7, which is activated by the NLRP3 inflammasome. Both N- and C-terminal fragments of the cleaved enzyme were extruded from chromatin at the transcription start sites (TSS) and decreased chromatin compaction, whereas NFκB recruitment and displacement were not affected by caspase 7 activity at the gene promoters (Figure 2, regulator scheme 2b). Similar observations were made during the differentiation of bone marrow-derived macrophages to osteoclasts, where auto-ADP-ribosylation of PARP1 primed the enzyme for NLRP3-dependent degradation [79].

PARP1 supports the gaining of a LPS-induced pro-inflammatory phenotype at the post-transcriptional level by stabilizing mRNA from pro-inflammatory genes including *Cxcl2* [70]. The PAR-synthesizing enzyme physically interacts with and poly-ADP-ribosylates RNA-binding protein HuR, which binds to elements rich in adenylate and uridylate (AU-rich elements) in target mRNAs and prevents them from degradation. Modification of HuR by PARP1 enhances nucleocytoplasmic shuttling and mRNA binding, hence promoting mRNA stability, whereas increases in the mRNA level or stability of pro-inflammatory cytokines/chemokines are abolished by PARP1 ablation or inhibition.

In conclusion, PARP1 promotes macrophage polarization towards the M1 phenotype at the transcription and post-transcription level by facilitating the expression of pro-inflammatory factors and stabilizing their mRNA.

### 6.3. The Role of PARP1 in STAT1/3 Signaling—Implications for Viral Infection

The immunomodulatory role of PARP1 was also documented upon viral infection, where PARP1 acts as both a pro- and antiviral factor in various cell types. This aspect was recently reviewed by Fehr and co-authors [80]. ADP-ribosylation facilitates the NFκB-dependent transcription of genes responsible for host‒pathogen interactions such as the previously mentioned *CCL2* cytokine, which acts as a chemoattractant for NK cells upon peritoneal infection with the vaccinia virus [72]. The defective NK cell recruitment and viral clearance in *Parp1 (-/-)* mice might not be solely caused by the PARP1 depletion in monocytes or macrophages. In any case, PARP1 can bind and ADP-ribosylate the replication and transcription activator (RTA) of gammaherpesviruses (γHVs), thereby inhibiting RTA’s ability to initiate lytic replication. In this context, PARP1 may possibly affect the preferential binding of RTA to tyrosine-phosphorylated STAT3 and indirectly modulate the transcription of STAT3-driven genes in response to γHVs infection [81]. Some viral proteins target PARP1 or make use of this enzyme to support pathogen replication. The processivity factor for DNA polymerase, PF-8 of human herpesvirus 6 and gammaherpesviruses, binds to PARP1 and targets it for ubiquitination and degradation, whereas the influenza A virus evades host innate immunity by PARP1-hemagglutinin-induced degradation of IFNAR1, a receptor for interferon alpha and beta. Viral invasion and replication, as well as the accumulation of DNA damage, give rise to cytosolic DNA that triggers the cGAS-STING-IRF3 signaling pathway and results in the transcription of pro-inflammatory genes and interferons, which are crucial for macrophage response. In *BRCA1*-deficient ovarian cancer cells, PARP1 inhibited antitumor immunity, whereas the inhibition of ADP-ribosylation increased the infiltration of antigen-presenting cells (including CD45+CD11b+MHCII+ macrophages characterized by pTBK1 and pIRF3) to pathological sites and led to STING-dependent increases in IFN-β and CXCL10 [82]. In addition to the external mode of macrophage polarization by inducing adjacent and distant cells to engage in the production and release of interferons, PARP1 was suggested to act as an activator of the cGAS-NF-κB pathway in CD11b+ macrophages and to drive their polarization toward the pro-inflammatory phenotype upon mice’s exposure to *Trypanosoma cruzi*-induced extracellular vesicles collected from the supernatants of infected cells [83]. The myocardial macrophage profile in Chagas disease showed the substantial reduction of TNF expression in *Parp2 (-/-)* versus *Parp1 (+/+)* macrophages. Furthermore, PARP1 controls transcription driven by STAT1, which transmits signals from the interferon receptors (Figure 2, regulator scheme 1). In a model of hyperglycemia-induced arteriosclerotic calcification, Parp1 promoted a pro-inflammatory phenotype of peritoneal macrophages by facilitating Stat1-dependent gene expression [84]. Interestingly, Parp1-deficient Raw237 macrophages and those isolated from *Parp1 (-/-)* mice exhibited a tendency to robustly develop into M2 macrophages instead of M1, due to the decreased transcriptional activity of Stat1. This was manifested in a decline of *iNOS* and enhanced *ARG1* transcription. Furthermore, the inhibition of ADP-ribosylation with PJ34 attenuated high glucose-induced macrophage calcification by inhibiting Runx2 expression and enhancing M2 differentiation. Moreover, IFN-γ and LPS/IFN-γ triggered the transcription of the *IL12b* gene encoding p40, a subunit of IL-12 and IL-23 in human peripheral blood mononuclear cells (PBMC), as well as in THP1-derived macrophages, in a PARP1-dependent fashion [85]. The enzyme recruitment to the *IL12b* locus resulted in promoter activation and p40 expression, whereas PARP1 silencing with shRNA resulted in the considerable reduction of p40 expression. This all leads to the conclusion that PARP1 promotes macrophage polarization towards the pro-inflammatory phenotype by regulating signaling triggered by IFN-γ and mediated by STAT1.

### 6.4. Extracellular Poly-ADP-ribose Polymers as Pro-inflammatory Ligands to Macrophage Surface Receptors

In the context of PARP1′s contribution to setting the pro-inflammatory phenotype of macrophages, one more signaling circuit emerges. Polymers of ADP-ribose and HMGB1 are released to the intracellular space by damaged cells, which undergo PARP1-induced parthanatos or necrosis. Both PAR and HMGB1 act as a “Damage-Associated Molecular Pattern” (DAMP) and drive inflammatory signaling (Figure 2, regulator scheme 2c), as reviewed by Pazzaglia et al. [45]. The PAR polymer is recognized by TLR2 and TLR4, much like bacterial pathogens, whereas HMGB1 is recognized by the receptor for advanced glycation end products (RAGE) and TLR4. Stimulation of TLRs induces the production of cytokines and chemokines in macrophages and furthers poly-ADP-ribosylation of HMGB1, which is secreted from cells. This pathway forms an amplification loop propelled by PAR-synthesizing enzymes in inflammation. Interestingly, some pathogens limit inflammation by interfering with the vicious cycle and cytokine signaling described above. For example, according to work by Pazzaglia and co-authors, *S. pyogenes* releases PARG to decompose free PAR polymers and gammaherpesviruses downregulates PARP1, whereas *Chlamydia trachomatis* causes the degradation of both DAMPs [45]. Summing up, PAR polymers released to the extracellular space during macrophage parthanatos may increase local inflammation by facilitating macrophage pro-inflammatory polarization. PARP activation by DNA damage or signaling cascades creates an additional pathway for macrophage pro-inflammatory specialization.

### 6.5. PARP1 in Anti-inflammatory Polarization of Macrophages—Insight into JAK-STAT Signaling and Nuclear Receptors

As for the anti-inflammatory response and phenotype, PARP1 is relatively highly abundant in human and mouse C57BL/6J macrophages and accounts for the majority of the PAR polymer synthesis. However, even in the M2(=LPS–)C57BL/6J-enriched signature, the enzyme seems to have a pro-inflammatory function, as reviewed by Orecchioni et al. [86]. On the other hand, in LPS-induced inflammatory macrophages, the PARP1 agonists repress *IL10*, an anti-inflammatory cytokine, whereas the relatively selective PARP1 inhibitor AG14361 was capable of increasing cytokine expression, which was even higher upon combined treatment with SIRT1 activator resveratrol [87]. Some anti-inflammatory action was also observed after a 4-h stimulation of RAW 264.7 cells and murine BMDMs with LPS, whereby PARP1 suppressed the transcription of *Il-6* via its interaction with MLL1 methyltransferase. Repression of *Il-6* occurred due to a decrease in the H3K4me3 marker at the NFκB binding site within the *Il-6* gene promoter, whereas PARP1 silencing and knockout resulted in a substantial increase in IL6 production [88].

Very little is known about the molecular contribution of PARP1 to the modulation of signaling cascades that lead to M2 macrophage polarization. The available data describe the external pathways associated with the expression of anti-inflammatory cytokines in other cell types. PARP1 positively regulates the production of Th1/Th2 cytokines such as IL-4/IL-5/GM-CSF and allergen-specific IgE in a mouse model of atopic dermatitis. Inhibition of PARP1 by olaparib treatment as well as *Parp1* gene knockout resulted in substantial alleviation of allergic symptoms [89]. Similarly, in response to anti-CD3 as well as anti-CD3 + anti-CD28 stimulation, T lymphocytes derived from *Parp1* (-/-) mice produced considerably less IL4, which triggers M2 polarization via JAK1/3-STAT6 signaling, when compared to their wild-type littermates [90]. Although direct evidence of PARP1 involvement in the JAK1/3-STAT6 cascade is missing, PARP1 enzymatic activity appeared to be required for STAT6 integrity in a murine model of allergen-induced inflammation [91]. The STAT6 protein was severely downregulated in spleens of PARP1-deficient mice, without any effect on mRNA levels. Neither PARP inhibitors exerted an effect on JAK1/JAK3 activation in response to IL-4 receptor stimulation. Furthermore, IL4-induced STAT6 drives chromatin remodeling and recruitment of retinoid x receptor (RXR) to de novo enhancers; thus, it triggers a secondary transcription factor wave, including peroxisome proliferator-activated receptor gamma (PPARγ), which forms PPARγ:RXR heterodimers (Figure 2, regulatory scheme 5). These are responsible for the appearance of ligand-preferred gene signatures in alternatively polarized macrophages [92]. RXR binds to thousands of sites in the genome, forming heterodimers with several different nuclear receptors, which act as genome-bound, ligand-dependent molecular switches on the predetermined landscape of macrophage enhancers occupied by lineage-determining transcription factors such as PU.1. PPARγ:RXR heterodimers mark lipid-sensitive enhancer sets and, upon stimulation with ligands, affect the expression of the corresponding genes and, thus, confer ligand-selective cellular responses and macrophage subtype specification (Figure 2, regulator scheme 4). In the extended signaling route from the IL4 receptor described above, PARP1 possibly acts as an antagonist of the M2 phenotype. Poly(ADP-ribosyl)ation has transcriptional repressor activity at the promoters controlled by RXR heterodimers, and PARP1 interacts directly with the DNA-binding domain of RXR on the response element, as reviewed by Morales and co-authors [93]. Similarly, PARylation of PPARγ by PARP1, which was confirmed by the transient silencing of the enzyme, prevents PPARγ binding to DNA and transactivation, reducing the transcription of PPAR gamma-target genes in a wide variety of cells [94]. This suggests that PARP1 and poly-ADP-ribosylation may inhibit the transcription of PPARγ:RXR-dependent genes. Furthermore, IL4-JAK1/JAK3-STAT6 signaling represses a large number of gene enhancers in a HDAC3-dependent manner during M2 polarization. The great majority of them overlap with the NF-κB p65 cistrome, exhibiting decreased responsiveness to lipopolysaccharide and activation of inflammasome after macrophage stimulation with IL4 [95].

Desgeorges et al. propose that the homeostatic release of glucocorticoids (GCs) after an inflammatory challenge plays an important protective role in returning the tissue and the organism to health [96]. After ligand binding, glucocorticoid receptors translocate from the cytoplasm to the nucleus, and (a) promote transcription by binding to corresponding glucocorticoid response elements (GREs) or (b) repress genes by directly interacting with DNA and by tethering transcription factors such as NFκB or AP-1. GCs inhibit the secretion of the pro-inflammatory cytokines TNFα, IL-1, and IL-6 in macrophages exposed to IFNγ, but stimulate monocytes to production of IL10 and TGFβ, inhibit transcription of *IL6* in macrophages, and, upon IL4-driven alternative polarization, stimulate secretion of fibronectin, thereby participating in matrix remodeling at the time of tissue repair. Upon HIV1 infection, PARP1 helps to prevent NFκB-dependent gene transcription by forming complexes with the HIV1 protein, Vpr, and the glucocorticoid receptor (Figure 2, regulator scheme 6) [97]. However, an interaction between GR and PARP1 was not observed after steroid (glucocorticoid) treatment.

Continuing with the context of alternative polarization, PARP1 controls the release of IL10 from various cell types, thereby promoting the M2 phenotype and anti-inflammatory response. As reviewed by Yelamos and co-authors, the enzyme negatively affects the development of regulatory T cells (Tregs) and T cell activation, which results in the production of VEGF, TGFβ, and IL-10 [98]. In patients with systemic lupus erythematosus, *PARP1* mRNA is targeted and silenced at the post-transcriptional level by miR-199-3p. This downregulation facilitates the activation of the ERK1/2 pathway and expression of IL10. Furthermore, clearance of infection-induced apoptotic cells by phagocytes suppresses autoimmune responses through the release of anti-inflammatory cytokines such as interleukin-10 (IL-10) and transforming growth factor-β (TGF-β), and the inhibition of pro-inflammatory cytokines [99]. PARP1 regulates the transcription of *IL10* in an allele-dependent way in macrophages engulfing apoptotic cells, but not upon LPS stimulation. It binds to -1082G>A alleles in the gene promoter, represses cytokine expression, and, thus, defines allele-dependent susceptibility to inflammatory pathology [85]. Furthermore, in cancer cells, PARP1 represses the anti-inflammatory cytokines *IL10* and *IL13* as well as *PD-L1*. The enzyme poly(ADP-ribosyl)ates STAT3, which transmits anti-inflammatory signals from the receptor for IL10 and promotes STAT3 dephosphorylation, which results in the reduced transcriptional activity of STAT3 (Figure 2, regulator scheme 7B) [100]. PARP1 silencing and pharmacologic inhibition of ADP-ribosylation with olaparib, veliparib, and A966492 suppressed the transcription of *PD-L1*. In *Parp1*-proficient wild mice, the IL6-STAT3-cyclin D1 pathway was active and augmented inflammation-driven colorectal tumor growth. On the contrary, expression of pro-inflammatory cytokines (IL-1β and IL-6), infiltration of CD11b (monocytes), F4/80 (macrophages), and STAT3 phosphorylation were substantially reduced in *Parp1 (−/−)* animals [101]. Similarly, in myocardial hypertrophy, PARP1 upregulated STAT3 transcriptional activity by retaining phosphorylated-STAT3 in the nucleus independently of JAK2 activation [102]. This was confirmed by the intramyocardial injection of adenovirus-encoding PARP1, which augmented cardiac hypertrophy in vivo, whereas PARP inhibition with 3-aminobenzamide attenuated the detrimental effect of ADP-ribosylation. Although the physical and functional interaction between PARP1 and STAT3 has not been described in macrophages, the role of the enzyme in STAT3-dependent gene transcription seems to be context-, stimuli-, or cell-type-specific, since such an interaction may up- or downregulate the activity of the transcription factor. Regardless of the direction of the STAT3 modulation, PARP1 in the above examples promoted pro-inflammatory cell response and brought STAT3 to enhance tissue injury.

In human monocytic cells, anti-inflammatory IL-10 selectively induces nuclear translocation and DNA-binding of p50-p50 homodimers, which play a critical role in the signal-specific transcriptional repression of pro-inflammatory genes that are transiently activated by p65‒p50 complexes (Figure 2, regulator scheme 7A) [103]. The NF-κB p50-p50 units recruit the transcriptional repressor histone deacetylase (HDAC)-1 to κB sites in regulatory regions of pro-inflammatory cytokines. Although the functional interaction between PARP1, p50, and EP300 was reported in the early 2000s, the conceivable involvement of PARP1 in the formation of repressive p50‒p50‒HDAC1 complexes has not been reported [104]. All these findings suggest that PARP1 limits the development of anti-inflammatory macrophages by repressing *IL10* in macrophages, but may promote the M2 phenotype by positively regulating the expression of anti-inflammatory cytokines in other cell types. The relatively numerous subset of M2 macrophages and the variety of extracellular and nuclear receptor ligands do not allow us to make an unequivocal statement about PARP1′s role in M2 specialization. Both intracellular pathways in particular M2 subsets and cytokine expression in adjacent cells must be taken into consideration.

### 6.6. PARP1 in the Metabolic Adjustment of a Macrophage to the Physiological Function—Cross-talk between Metabolism and ROS Production

One more aspect of the metabolic signature that is relevant to macrophage polarization needs to be mentioned. The gain of a particular phenotype is associated with the reprogramming of pathways that fuel phagocytes. Alternatively activated macrophages make use of fatty acids and increase the mitochondrial respiratory capacity, while M1 macrophages preferentially derive ATP from glycolysis. IL-4 upregulates OXPHOS via the transcription factor STAT6 and PPARγ coactivator-1β (PGC-1β), the overexpression of which reduces the production of pro-inflammatory cytokines, as reviewed by Thapa et al. [105]. IL-10 also drives anti-inflammatory polarization by stimulation of OXPHOS and metabolic reprogramming of macrophages [106]. IL10 suppresses glycolysis in LPS-stimulated bone-marrow-derived macrophages. In contrast, LPS and other pro-inflammatory stimuli enhance aerobic glycolysis in these phagocytes. In bone-marrow-derived macrophages isolated from *Parp1* (-/-) mice, the NAD+ level was rescued upon LPS stimulation and was responsible for the observed metabolic shift to aerobic glycolysis, also known as the Warburg effect [9]. According to a review by Diskin et al., the consumption of NAD+, which serves as a cofactor of numerous metabolically relevant enzymes, upon PARP1 activation affects the activity of these enzymes that utilize NAD+ as a substrate. Some of them control the transcription of numerous proteins involved in ATP production [107]. For instance, NAD+ depletion leads to the inactivation of deacetylase SIRT1, which, in turn, influences the protein targets involved in metabolic regulation such as PPARs, autophagy-related proteins (Atg), sterol regulatory element-binding protein (SREBP), and cAMP response element-binding protein (CREB), as well as modulates gene transcription by preventing nucleosome deacetylation (reviewed by Ke et al.) [108]. As reviewed by Szántó et al., the expression of fatty acid transporters FABP7, FABP3, CD36, and aP2 (FABP4) is regulated by PARP1, whereas multiple steps in the tricarboxylic acid cycle (TCA) require NADH for the transition from one step to another [109]. PARP inhibition with ABT-888 and BMN-673 improves mitochondrial function and energy balance [110]. In cortical neurons, the increase in hexokinase activity subject to MNNG treatment was reversed by PARP inhibitor DPQ [111]. This suggests that ADP-ribosylation impedes the glycolytic flux by inhibiting hexokinase 1 independent of NAD+ depletion, PARylating and, hence, inhibiting glyceraldehyde-3-phosphate dehydrogenase. Subunits of pyruvate dehydrogenase complex (PDPR, PDHA1, PDHX) also undergo poly-ADP-ribosylation [109]. Only these few examples indicate the detrimental role of the enzyme in the outcome of metabolic routs, which might be of particular importance for the modulation or maintaining of the macrophage phenotype. The breaks in the TCA cycle lead to the accumulation of itaconate, which acts as a microbicide compound, and succinate, which activates the transcription of genes involved in glycolysis via HIF1α and pro-inflammatory cytokines. PARP1 co-activates HIF-1alpha-dependent gene expression by forming a complex with HIF-1alpha through direct protein interaction [112]. PARP1 silencing with siRNA in the myelogenous leukemia cell line K562 considerably reduced HIF-1-dependent gene expression. Cell treatment with PARP inhibitor DAM-TIQ-A further confirmed that HIF-1 ADP-ribosylation is crucial to its transcriptional activity.

Pro-inflammatory macrophages are characterized by the intensification of the pentose phosphate pathway (PPP), which generates pentoses and 5-ribose phosphate for nucleic acid production but also serves as the major source of NADPH. This is utilized as the reducing power required for glutathione and thioredoxin reduction, thereby allowing for the clearance of harmful ROS, but also serving as a substrate for NADPH oxidase. It catalyzes the generation of superoxide anion, that is then converted to a variety of oxidants capable of pathogen killing [107]. Bone-marrow-derived monocytes, and then macrophages, are documented to express two NOX isoforms: NOX2 and NOX4. In mouse kidneys, the inhibition of PARP1 with AIQ and PJ34 inhibitors as well as PARP1 gene deletion reduced cisplatin-induced leukocyte and macrophage infiltration, myeloperoxidase activity, expression of adhesion molecules, pro-inflammatory cytokines (TNFα, IL1β), and both NOXes [113]. However, in some cancer cell types, ADP-ribosylation emerged as an repressor of *NOX1* and *NOX4* since cell treatment with PJ34 resulted in upregulation of *NOX1* and *NOX4* mRNAs [114]. The latter enzyme plays a role in defining the macrophage phenotype and the direction of their polarization [115]. NOX4 promotes the development of anti-inflammatory phagocytes by the induction of STAT6 and, as a consequence, the reduction of NFκB activity. The deficiency of NOX4 promotes M1 polarization and enhances NOX2 expression in response to cell stimulation with LPS+IFNγ, which results in a further increase in the production of a superoxide anion. Thus, *NOX4* repression by ADP-ribosylation may foster the pro-inflammatory phenotype of macrophages. Furthermore, the cleavage of PARP1 in response to endotoxic shock and to intestinal and renal ischemia reperfusions resulted in an increase in NFκB-dependent transcription of inducible nitric oxide synthase (*NOS2*), which was followed by intensive production of nitric oxide, and in the increase in the activity of myeloperoxidase [116]. Similarly to *Il6*, and also to the promoter of *Nos2*, PARP1 impedes the access of chromatin to canonical NFκB heterodimers and reduces gene transcription upon TLR stimulation. The promoter’s resistance to NFκB signaling is reversed by the activation of NLRC4-caspase 1 inflammasome in response to, inter alia, bacterial ligands of TLRs in murine BMDMs [117]. The inflammasome cleaves PARP1 and allows for NFκB recruitment to *Nos2* promoters, whereas macrophages isolated from mice that express Parp1^D214N^ cleavage-resistant mutants produce considerably less Nos2 upon TLR stimulation. Both nitric oxide and hydrogen peroxide are membrane-permeable and, in addition to pathogen killing in phagosomes, act as intra- and cell-to-cell signaling molecules promoting cytokine release, and as activators of adjacent cells [118]. Interestingly, human monocyte-derived M2 macrophages also require ROS to acquire anti-inflammatory and protumorigenic phenotypes during IL-4-stimulated polarization, and intracellular hydrogen peroxide is responsible for increased M2 markers [66]. Interestingly, the transcription of *NOX2*, *NOX5*, and the nonenzymatic members of the NOX complexes, *p22phox* and *p47phox*, is lower when compared to M1 macrophages.

In conclusion, LPS-induced repression and cleavage of PARP1 possibly facilitate the gaining of the pro-inflammatory phenotype by rescuing the intracellular level of NAD+ as well as increasing the transcription of *NOX*es and *NOS2*. The enzyme control activity of enzymes is involved in the key metabolic pathways and expression of transporters for metabolic substrates.

### 6.7. The Role of PARP1 in Defining Enzymatic Defense Against Oxidative Condition in Polarized Macrophages

The shift to the pro-oxidative condition during macrophage polarization is counteracted by the increased expression of enzymes that defend cells from oxidative injuries. M2 macrophages are characterized by higher levels of antioxidant enzymes Cu/ZnSOD (SOD1), glutathione peroxidase 1 (Gpx1), and catalase (Cat), whereas M1 is characterized by MnSOD (SOD2), glutathione reductase (Gsr), thioredoxin reductase 1 (Txnrd1), and peroxiredoxin (Prdx1). In LPS-induced pro-inflammatory macrophages, the transcription of *CAT* and *Cu/ZnSOD* is considerably decreased. In cisplatin-induced injury to the kidney proximal tubule epithelial cells, PARylation suppressed transcription of *MnSOD* and *CAT* by repressing *SIRT3* [119]. Similarly, in M1 macrophages, PARP1 served as an inhibitor of *MnSOD* transcription, and PARP1 displacement from the *MnSOD* promoter induced by LPS allowed for transcriptional gene activation by canonical NFκB [8,9]. Importantly, *PARP1* repression in response to LPS in both human and mouse macrophages rendered these cells resistant to exogenous oxidative stress by *MnSOD* release from PARP1-dependent silencing. Nothing is known about the contribution of PARP1 to the regulation of other antioxidant enzymes, which are controlled by NFκB (glutathione S-transferase, metallothionein-3, NAD(P)H dehydrogenase [quinone]1, heme oxygenase-1, and glutathione peroxidase-1), as reviewed by Lingappan [120]. The observed repression of enzymes that immediately convert the two reactive oxygen species, superoxide anion and hydrogen peroxide, may facilitate NFκB transcriptional activity in pro-inflammatory macrophages. Furthermore, NFκB antagonizes the activity of nuclear factor erythroid 2 (NFE2)-related factor 2 (NRF2) by competing for the transcriptional co-activator CBP (CREB-binding protein)–p300 complex. NRF2 dissociates from its repressor Keap1, translocates to the nucleus in response to a physiological shift towards oxidant production, and activates the transcription of enzymes involved in the scavenging of electrophiles (NAD(P)H quinone oxidoreductase 1, heme oxygenase-1; aldo-keto reductase family 1, member C1, Mn/Zn superoxide dismutase), secondary metabolites (phase II detoxifying enzymes such as glutathione S-transferase, UDP-glucuronosyltransferase, catalytic and modifier subunits of glutamate cysteine ligase), and drug transporters (multidrug resistance-associated proteins) [121]. Cancer cell-derived lactate was shown to activate NRF2 and, thereby, skews macrophages’ polarization towards an M2-like phenotype [122]. PARP1 knockdown with siRNA suppresses NRF2-dependent gene transcription, suggesting that the full transcriptional activity of this transcription factor requires PARP1. The enzyme binds to antioxidant response element (ARE) at the promoters of Nrf2 target genes, interacts directly with small Maf proteins, and enhances the interaction among Nrf2, MafG, and ARE [123]. Furthermore, NRF2 (similarly to PARP1) interferes with *IL6* induction and, thus, with inflammatory phenotypes in vivo [124]. The unknown mechanism of some gene expression in pro-inflammatory macrophages may involve PARP1‒MAFG‒NRF2 complexes, but such an idea needs to be experimentally confirmed. Furthermore, according to the review by Wang et al., FOXO3 protects against oxidative stress by increasing the transcription of *MnSOD*, *CAT*, and peroxiredoxin III (*PRX3*), and is also critical to hematopoietic self-renewal [125]. In the macrophages of old mice, the decrease in FOXO3 protein with age caused a loss of anti-inflammatory phenotype, whereas the PI3K/Akt/FOXO3 axis triggered by *Mycobacterium tuberculosis* suppressed transcription of *IL10* and promoted a pro-inflammatory response in phagocytes, as reviewed by Stefanetti et al. [126,127]. FOXO3 was reported as a target for PARP1-mediated ADP-ribosylation, but the role of this modification in FOXO3 transcriptional activity is contradictory in the two available papers. The lack of any direct evidence of PARP1‒FOXO3 interaction in macrophages does not allow us to estimate to what extent such a possible cascade affects the expression of antioxidant enzymes and drives macrophage polarization. In conclusion, PARP1 repression during macrophage polarization towards the M1 phenotype may provide the required protection against reactive oxidants by allowing for higher expression of genes involved in the ROS removal. Furthermore, the lower PARP1 abundance may also limit NFR2 activity, which prevents the gaining of the pro-inflammatory phenotype.

Protection of the genome is provided by DNA repair pathways, which are particularly effective in macrophages due to the high transcription of genes involved in recognition, removal of lesions, gap filling, and ligation [128]. When compared to monocytes, macrophages express higher levels of *XRCC1*, *PARP1*, and *Lig IIIa*, which are involved in BER and HR. This makes macrophages less prone to the damage caused by oxidative agents. Similarly to *PARP1*, the transcription of the subset of genes involved in DNA protection is controlled by cell cycle progression and is considerably increased in proliferating macrophages. A recent study revealed that EP300‒BRG1 complexes, which were found in the promoters of DNA repair genes in macrophages, also operate in cancer cells and control the expression of the same group of functionally linked genes. Importantly, in the studied breast cells the transcription yield from some EP300‒BRG1-driven promoters was tuned by the occurrence of PARP1 and ADP-ribosylation of EP300. Inhibition of PARylation and transient PARP1 silencing substantially reduced the transcription of some genes involved in DNA repair, such as *BRCA1/2*, *LIG1*, and *NEIL3*. ADP-ribosylation of the acetyltransferase enhanced the enzyme activity and acetylation of the nucleosome. In that model, PARP1 served as a co-activator of cell cycle-dependent genes. The uniform mode of transcription regulation at the promoters characterized by E2F motifs and CpG islands in different cell types allows us to speculate that PARP1 may also occur at cell cycle-dependent gene promoters in macrophages and enhance the transcription of genes relevant to DNA repair. Therefore, PARP1 may facilitate the removal of DNA lesions dually: directly, by acting as a component of repair machineries; and indirectly, by increasing the expression of their constituents. Again, the possible indirect fashion of PARP1 contribution to DNA repair systems must be confirmed experimentally.

## 7. PARP1 Inhibitors in the Intentional Modulation of Monocyte and Macrophage Responses

### 7.1. Possible Benefits of PARP Inhibitors in the Treatment of Inflammatory-Relevant Disorders

PARP inhibitors that are under clinical assessment mostly compete with NAD+ for PARP1 and PARP2 and prevent the formation of poly(ADPribose) polymers. However, compounds such as ME0328 decrease the activity of PARP1 and PARP3, whereas several others including rucaparib, PJ34, and 3-aminobenzamide act as pan-PARP inhibitors. In addition to catalytic inhibition, some compounds are capable of PARP1 trapping and promote the formation of PARP1‒DNA complexes. According to Murai and Pommier, their trapping potency can be ranked as follows: talazoparib >> niraparib ~ olaparib ~ rucaparib > veliparib [129]. The development of the two groups of inhibitors extends the number of options for their use in intervention in intracellular processes. An example of the rationale for choosing between different PARP inhibitors comes from anticancer approaches, where PARP trapping and PARylation inhibition or the latter alone accounted for the synergy with alkylating agents or Top1 inhibitors. Very recently, one more group of PARP-targeting agents was developed, namely small molecule PARP degraders. One such compound, iRucaparib-AP6, mimics PARP1 genetic depletion, blocking both the catalytic activity and scaffolding effects of PARP1 [130]. In monocytes and macrophages, as well as in their precursors, the reduction of protein PARylation and PARP1 level, as well as PARP1 maintenance on the chromatin, may allow for control of at least some innate immune responses. By fueling inflammation, activation of the enzyme promotes colitis, rheumatoid arthritis, multiple sclerosis, diabetes, neurodegenerative disorders (including Parkinson’s and Alzheimer’s disease), infarction‒reperfusion, and septic shock [45]. PARP1 plays a pivotal role in this vicious cycle by interconnecting amplification of DNA damage, inflammation, and cell necrosis during degenerative processes that can induce further activation of PARP-1 and release of poly-ADP-ribose to the intracellular space [45]. Therefore, PARP inhibitors may quench the inflammation triggered by PAR that is released from damaged and dying cells during tissue injury. On the one hand, DAMPs facilitate sterile inflammation, which is important for tissue repair and regeneration, but on the other hand, they lead to the development of numerous inflammatory diseases, such as metabolic disorders, neurodegenerative diseases, autoimmune diseases, and cancer, as reviewed by Gong et al. [131].

Another option in targeting PARP1-dependent cascades or transcription that require enzyme cleavage is provided by the NLRP3 inflammasome. Inhibition of caspases, particularly caspase-1 and caspase-3/7, was shown to downregulate the transcription of pro-inflammatory cytokine subset and GM-CSF upon TRL stimulation with LPS [78]. Such an attempt to curtail M1 activity can act dually: it can prevent the expression of genes that undergo activation upon PARP1 fragmentation, and the release of cytokines IL-1β and IL-18, transcription of which is considerably enhanced by the enzyme. On the other hand, IL-18 is capable of suppression of Th17 cell differentiation and elevation of Foxp3+ Treg cells, hence reducing inflammation. Similarly, Xu et al. showed that the inflammasome plays opposing roles in cancer development due to different immune responses, but in some cancer types inflammasome-induced excessive inflammation acts as a detrimental factor and inflammasome inhibitors are considered a promising approach for cancer prevention and treatment [132].

### 7.2. Specificity and Off-Targets of ADP-Ribosylation Inhibitors

Although the examples listed above agree on the possible beneficial effects of PAR synthesis inhibitors in the treatment of inflammatory-relevant disorders as well as some parasitic and viral infections, there is scant evidence of the contribution of other PARPs to macrophage physiology. PARP1, PARP2, PARP9, and PARP14 were shown to promote or suppress macrophage responses to stimuli. The transcription of other family members such as PARP3, PARP4, PARP7, PARP8, PARP10, PARP11, PARP12, and PARP13 increased upon TLR activation with LPS in murine bone-marrow-derived macrophages [80]. These proteins may also shape macrophage polarization. For example, PARP12 was identified as an interferon-induced gene playing a potential role in cellular defenses against viral infections; its association with p62/SQSTM1 correlates with increased NF-kB signaling, suggesting the contribution of PARP12 to inflammation. PARP10 also acts in the NF-kB cascade and ADP-ribosylates NEMO thereby preventing activation of IKK and inhibiting pro-inflammatory response. PARP7 prevents phosphorylation of IRF3 by ADP-ribosylating TBK1. Furthermore, PARP13 binds to IFN mRNA and targets it for degradation, whereas PARP11 targets IFNAR for proteasome-dependent degradation by ADP-ribosylating the E3 ubiquitin ligase β-TrCP. More examples on the contribution of other PARPs in the inflammatory responses can be found in the review paper by Fehr et al. [80]. Since most of the reported interactions are mediated by ADP-ribosylation, the use of pan-PARP inhibitors possibly interferes with signaling pathways at different levels and results in the effects opposite to only PARP1 inhibition.

Furthermore, due to the high conservation of the catalytic domain across PARPs, the current inhibitors of PARylation affect the activity of other enzymes such as PARP2. For example, olaparib, which was originally described as a PARP1 and 2 inhibitor, has recently been shown to be a potent PARP3 inhibitor; despite the “monoenzymatic” activity of PARP3 being similar to that of other mono(ADP-ribosyl) transferases (MARTs), PARP-4, PARP-6, PARP-10, PARP-14, PARP-15, and PARP-16 transfer a single ADP-ribose unit to target proteins [133]. Hence, PARP-1‒3 inhibitors may possibly affect the activity of other PARPs and MARTs. A more comprehensive understanding of the role of particular PARPs in PARylation- and MARylation-dependent macrophage activation and inflammatory diseases is needed to selectively target PARP1 activity with the currently available PARP inhibitors. PARP1′s contribution to HIV infection may serve as an example of functional antagonism in the PARP family since other members mostly demonstrated antiviral activity, in contrast to PARP1. Thus, targeting PARP1 activity with pan-PARP inhibitors may lead to adverse effects. Another problem with PARP1 inhibition results from the fact that even a small amount of poly-ADP-ribose polymers, which are characterized by a relatively low expression of the enzyme, in cells might be critical to their survival or proper functioning, and the risk‒benefit ratio might be too high for repurposing the currently available inhibitors for nononcological indications. More information regarding the latter aspect can be found in Berger et al. [134]. The synthesis of highly selective PARP1 inhibitors or the specific enzyme degradation with small-molecule PARP1 degraders may resolve issues related to off-target effects. The search for poly-ADP-ribosylation-dependent intracellular cascades that are specific to the particular cell type or tissue can also limit the toxicity of PARP inhibitors in other parts of the body. In monocytes and macrophages, the possible functional interaction between PARP1 and lineage-determining factor PU.1 can be taken into consideration to intentionally modulate transcription of the monocyte and macrophage-specific gene enhancers. The identification of more cell type-restricted factors that operate in a PAR-dependent fashion and control the development of particular macrophage subtypes is needed.

### 7.3. PARP‒DNA Traps in the Modulation of Monocyte and Macrophage Responses

Although the same concerns apply to PARP poisons, which cause the formation of PARP1‒DNA complexes, they give another option to modulate the immune response. Only 2% of PARP1 proteins are associated with chromatin, but this is sufficient to control the transcription of some genes. The specific sequence that is recognized by the enzyme and the set of genes transcriptionally controlled by PARP1 remains unknown. Recent data from cancer cells revealed the enzyme preference for cell cycle-dependent promoters characterized by the E2F motifs, the presence of CpG islands, and the occurrence of BRG1-based SWI/SNF complexes, but PARP1 acted as an activator once all these features were followed by histone acetylation [135]. The long list of PARP1-BRG1-H3K27ac-E2F-CpG promoters comprises functionally related genes responsible for, inter alia, DNA repair, cell cycle progression, and the response to various stimuli including oxidative stress, metabolism, nitrogen compound, signaling, and gene transcription. Since the cell cycle progression defines the transcription rate from BRG1-H3K27ac-E2F-CpG promoters (where the H3K27ac status is regulated by the ratio between the activity of EP300 and HDAC1), in proliferating macrophages some of genes encoding proteins involved in DNA repair are also regulated accordingly [10]. Nevertheless, the role of PARP1 in the described mechanism in monocytes or macrophages has not been confirmed yet. The abovementioned regulatory units operate in a poly-ADP-ribosylation-dependent mode in cancer cells; thus, olaparib and PARP1 silencing successfully interfered with the gene transcription. However, some gene promoters that were controlled by poly-APD-ribosylation emerged, dependent on PARP1 occurrence and the physical interaction of the enzyme with the chromatin upon macrophage stimulation with LPS. An example of the shift in PARP1-dependent transcription control of *SOD2* has been recently documented by Tokarz et al. [8]. Thus, PARP traps can be considered as molecular tools capable of protecting pro-inflammatory macrophages from oxidative stress-induced death. In light of these results, a further search for genes transcriptionally controlled by PARP1 shuttling to and from the gene regulatory regions may extend the list of known and possible targets for intervention in macrophage polarization or development. If the genomic distribution of PARP1‒BRG1‒EP300 complexes is similar in proliferating cancer cells and macrophages, then interference with PARP1 displacement may impact the intracellular processes listed in the abovementioned paper by Sobczak et al. Again, the molecular switch that makes the transcription machinery dependent on PARP1 physical interaction with chromatin or poly-ADP-ribosylation of co-operating enzymes BRG1 and EP300 remains unknown. Another example of gene control by PARP1′s occurrence at the gene promoters comes from Robaszkiewicz et al., who found that, in growth-arrested monocytes, the transcription of some surface receptors and signaling mediators downstream of TLRs were suppressed by the assembly of RBL2-based repressive complexes and PARP1 deficiency [48]. The identification of other chromatin-remodeling enzymes, which operate in the same signaling circuit, and the use of their inhibitors, activators, or traps may mimic the effect of PARP1 at the promoter gene driven by the PAR-synthesizing enzyme. Furthermore, the intentional suppression of genes that are simultaneously activated by PARP1 and cell proliferation can be achieved in macrophages by inhibition of cyclin-dependent kinases, which prevent cell cycle progression from G1 to S phase and, thereby, suppress the expression of PARP1. The efficiency of CDK4/6 inhibitors in the suppression of some DNA repair genes in monocyte-derived macrophages was experimentally confirmed [10]. Moreover, numerous such compounds are being tested as anticancer drugs in clinical trials; hence, information on toxicity, side effects, and therapeutically relevant doses has been collected. On the one hand, such an approach provides some specificity in terms of cell types and targets PARP1-dependent gene expression only in proliferating cells such as macrophages. On the other hand, it limits target gene specificity since the cell arrest in G1 reduces most E2F-driven genes and deprives cells of numerous proteins. In any case, the understanding of the molecular mechanism behind PARP1 occurrence on the chromatin and the mode of transcription control may allow us to modulate the transcription of some gene subsets with PARP‒DNA traps, particularly when the gene transcription is up- or downregulated by PARP1 at the gene regulatory elements.

### 7.4. PARP Inhibitors as Possible Guardians of Hematopoietic Stem Cell Pluripotency

One more option to consider is the application of PARP1 inhibitors and traps in the expansion and maintenance of embryonic stem cells and hematopoietic precursors of monocytes and macrophages, as well as in the intentional induction of the cell commitment. Since PARP1 occupies promoters of pluripotency genes, the PARP poisons may be used to support self-renewal and stemness in the culture. More information is needed on the potential role of PARP1 decline or eviction from the abovementioned gene promoters during stem cell senescence or spontaneous lineage commitment. Moreover, modulators of bone marrow kinetics play a prominent role in the defining of damage and recovery patterns after injury. The classical role of PARP1 in the maintenance of mouse hematopoietic stem cells and human lymphoblasts was shown in a model of salidroside-induced protection against oxidative stress, where glycoside activated poly-ADP-ribosylation and base excision repair and, thereby, prevented apoptosis and self-renewal defects in transplanted recipients. As expected, PARP1 inhibition with NU1025 allowed for the recruitment of quiescent HSCs into cell cycling on in vivo challenge with oxidative stress [136]. Although an increase in the rate of HSC divisions may counteract some acute damage by enhancing regeneration and shortening the duration of blood aplasia, the stimulation of HSC divisions may exhaust the stem cell pool and increase the severity of late effects. Therefore, PARP inhibitors can also be considered as guardians of stem cell reservoirs. In the culture of primary bone marrow mononuclear cells collected from patients with myelodysplastic syndromes, olaparib revealed toxicity against myeloid cells, causing a significant reduction of blasts and promyelocytes, but increased the number of metamyelocytes and mature granulocytes by promoting a dose-dependent increase of PU.1 and CEBPA transcription factors, which drive the differentiation of granulocytes and monocytes [137]. In line with these findings is another set of data from the culture of *BCR‒ABL1*-positive HSC, which lost repopulation activity upon PARP inhibition [138]. However, the latter study applies to a *BRCA*-defective background and the observed decrease in proliferation potential might result from the impaired effectiveness of homologous recombination rather than interference with the decrease in stemness potential. The reports that describe the potential of PARP inhibitors in other types of stem cells confirm the general hypothesis on the possible use of these drugs for interference or maintaining pluripotency. For example, in a model of brain trauma, the PARP inhibitor PJ34 significantly improved the effectiveness of neural stem cell transplantation and promoted rapid functional recovery PARP1 trapping at the regulatory elements of genes that drive cell pluripotency and self-renewal potential as well as inhibition of ADP-ribosylation may maintain features of monocyte and macrophage precursors and limit the number of differentiated cells, thereby providing another approach to interfere with immune responses. However, such a hypothesis needs experimental verification.

### 7.5. PARP Inhibitors in the Direct Treatment of Hematopoietic Cancers

In addition to the FDA-approved approach to treat some *BRCA1*-deficient breast and ovary cancers with PARP inhibitors, these drugs have emerged as a potential cure that acts directly on hematopoietic cancers. Acute myeloid leukemia (AML) is the most common type of leukemia and is characterized by a poor prognosis. Similar to other malignant cancer types, AML is a highly proliferative and highly invasive type of tumor. Nowadays, about 35‒40% of adults aged 60 or under can be cured, while in older patients, only 5‒15% might recover, as described in a review by Döhner et al. [139]. According to data presented by the American Cancer Society, 21,450 new cases of AML were registered in the USA in 2019. Moreover, given that 11,650 patients died of AML, this type of cancer emerged as the deadliest type of leukemia in the USA in the previous year [140]. The lethal phenotype of AML results from the heterogeneity of genes frequently mutated in this tumor, which encode either the most crucial components of cell survival and proliferation cellular pathways or their epigenetic regulators. These comprise *RUNX1* transcription factor, one of the most commonly mutated marker genes in AML, as reviewed by Döhner et al. [139]. Leukemic cells that carry *RUNX1* gene abnormalities are characterized by an undifferentiated phenotype. RUNX1 expression negatively correlates with PARP1 activity in undifferentiated stem cells. In RUNX1‒RUNX1T1-transformed murine AML cells, PARP inhibitors triggered differentiation into monocytic and granulocytic cells [141]. This suggests the possibility of driving undifferentiated, highly proliferative AML cells into more stable, differentiated phenotypes with PARP inhibitors. Another mutation in the gene encoding the FTL3 kinase receptor involved in RAS-RAF, PI3K-AKT, and JAK-STAT signaling is crucial to AML development. It regulates the proliferation and survival of the cancer. High expression of PARP1 in AML patients correlates with a poor prognosis, and with the frequency of mutations in *FTL3-ITD* and DNA (cytosine-5)-methyltransferase 3A (*DNMT3a*) [142].

The most crucial activity of PARP1, not only in myeloid lineage malignancies but in cancer cells in general, encompasses its involvement in base excision repair (BER). For this reason, poly (ADP-ribose) polymerase 1 is considered a potential therapeutic target in anticancer therapies. One of the most interesting concepts in modern oncology is synthetic lethality, an approach that makes use of the weak points of some cancer phenotypes. PARP inhibitors have been reported as useful tools in triggering the synthetic lethality in a *BRCA1*- or *BRCA2*-deficient background of breast and ovarian cancer. According to Huang et al., such cells are severely impaired in mechanisms responsible for the removal of DNA damage [143]. Importantly, decreased levels of BRCA1 expression were detected in a fraction of AML patient-derived cells as well. *BRCA1* repression correlates with its promoter hypermethylation and a slight increase in *DNMT3A* transcription [144]. Therefore, in *BRCA*-deficient AML, PARP inhibitors may be considered an ideal solution to further prevent removal of DNA lesions and potentially increase the efficacy of DNA-damaging chemotherapeutic drugs, which are commonly used in anticancer therapies. According to the DrugBank database, the four PARP inhibitors, niraparib, olaparib, rucaparib, and talazoparib, have been approved by the FDA for the treatment of oncological patients. As mentioned above, PARP inhibitors reach their peak efficiency in *BRCA*-deficient cells. The mechanism of cytotoxic action of niraparib, olaparib, and rucaparib involves the formation of a PARP1‒DNA complex, while talazoparib is a steric inhibitor of the NAD+ binding domain [145]. Notably, PARP1 contributes to epigenetic regulation and controls the transcription of genes that are crucial for tumor survival and progression due to their involvement in proliferation, DNA repair, metabolism, and many other processes. The enzyme does so by interacting with chromatin-remodeling enzymes such as histone acetytransferase EP300, the activity of which is regulated by PARylation in breast cancer cells [42]. PARP inhibitors increase the efficiency of some inhibitors of epigenetic writers and erasers. One of the standard strategies to fight myeloid-line malignancies includes inhibitors of DNA methyltranferases (DNMTs), which are known for their involvement in the initiation of AML. Previously, the FDA had only approved two DNMT inhibitors for clinical use, azacytidine (5-azaC) and decitabine (5-aza-dC). Azacytidine, after metabolic changes, can be incorporated in both RNA and DNA, while decitabine can only be incorporated into double-stranded DNA. The mechanism of action of these cytosine analogs, as described by Ganesan et al. and Gnyszka et al. in their reviews, includes entrapment of the DNA methyltransferase and preventing it from further DNA methylation [146,147]. Upon DNA damage, DNMTs and PARP1 are recruited to the lesion. Azacytidine and decitabine enhance the PARP1 chromatin immobilization caused by talazoparib. Moreover, the combination of these drugs led to an increased frequency of double-strand breaks (DSBs) in AML and improved animal survivability in murine xenograft models [148]. A similar effect was observed in the culture of AML cells treated with a combination of niraparib, decitabine, and the HDAC inhibitor romidepsin. The three drugs synergistically decreased proliferation, induced apoptosis, and trapped DNMT1 and PARP1 to chromatin. Two alkylating agents, melfalan and busulfan, intensified the antiproliferative and proapoptotic effects of the combination treatment [149]. Talazoparib, in combination with the HDAC inhibitor SAHA and the alkylating agent bendamustine (also known as NL101), substantially inhibited the growth of AML cells by arresting them in G2/M, and led to an accumulation of DNA breaks. The efficiency of the described combination of drugs was also confirmed in vivo, where these compounds prolonged the survival of mice in a xenograft model [142].

Despite the growing attention paid to PARP1 in the past two decades, a fully effective treatment for cancer-diagnosed patients has not been discovered yet. PARP inhibitors still undergo extensive testing and clinical trials, not only as monotherapies in the previously described BRCAness phenotype, but also as an additive to standard chemotherapy. According to ClinicalTrials.gov, there are two active phase I studies and one phase II study currently recruiting patients dedicated to testing the anticancer potential of PARP inhibitor veliparib in combination with other common chemotherapeutics (carboplatin, topotecan hydrochloride, temozolomide) in various types of leukemia, including AML.

In addition to involvement in DNA repair and proliferation, PARP1 participates in the immunomodulatory processes of mononuclear cancer cells. Its impact on the immune response in healthy human or murine myeloid cells was described thoroughly in the foregoing paragraphs. Worldwide, many studies are being conducted on leukemic cells, which due to their distinct physiological properties cannot be compared with healthy cells. Despite the phenotypic differences between healthy and leukemic mononuclear cells, PARP1 seems to regulate comparable pathways and processes, particularly in proinflammatory responses. PARP1 expression, as well as the level of protein PARylation that usually corresponds to the PARP1 activity, is known to increase the response to proinflammatory stimuli such as LPS or cytokines in cancer cells. PARP1 deficiency considerably reduced the NFκB-dependent transcription of cytokines such as *IL-1β* and *IL-18*, thereby suggesting that PARP inhibitors reduce the local level of proinflammatory factors in the environment of leukemic cells [70]. Activation of the enzyme is induced by phosphorylation carried out by ERK1/2 in the aforementioned ERK1/2‒PARP1‒NFκB pathway triggered by bacterial patterns. Another protein kinase that appears to be involved in PARP1 phosphorylation and, therefore, in the regulation of the inflammatory response in leukemic cells, is c-Abl. Physical interaction and phosphorylation of PARP1 by c-Abl tyrosine kinase in leukemic RAW 264.7 and THP1 cells is crucial for the activation of the proinflammatory NFκB pathway and the transcription of cytokines such as *TNFα* and *IL1β* [150]. Besides its immunomodulatory role, NFκB contributes to DNA damage repair in AML cells through transcriptional regulation of PARP1 at the enzyme promoter. RelA/p65 knockdown resulted in the accumulation of double-strand breaks (DSB) in comparison to cells that were transfected with an empty vector. Moreover, p65-deficient AML cells were characterized by decreased mRNA and protein levels of PARP1. Interestingly, knockdown of both PARP1 and RELA resulted in more robust damage accumulation in comparison to knockdown of either of these separately [151]. RELA may indirectly influence PARP1 activity through regulating uc002jit.1, long noncoding RNA (lncRNA) that is thought to stabilize the mRNA of PARP1, as well as prevent it from degradation in the cytoplasm. Upon its knockdown, the enzymatic action of PARP1 and the proliferation of leukemic cells were drastically limited. Moreover, uc002jit.1-deficient leukemic cells were more susceptible to chemotherapy [152]. Taking into consideration the interdependence between PARP1 and NFκB, it is possible that a combination of PARP and NFκB inhibitors may lead to even higher accumulation of DNA damage in cancer cells and silencing of proinflammatory cascades. The numerous intracellular processes that are positively regulated by PARP1 and poly-ADP-ribosylation allow us to speculate on the possible beneficial effects of PARP inhibitors in treating hematopoietic malignancies. Further attempts to develop specific PARP1 inhibitors should discriminate between processes controlled by the various PARP family members to provide the expected cure for AML.

### 7.6. PARP Inhibitors in Anticancer Immunotherapy

The described immunomodulatory activities of PARP1 may serve as a starting point for anticancer immunotherapy. A relatively new anticancer strategy involves targeting tumor associated-macrophages (TAMs), which infiltrate the tumor microenvironment, stimulate tumor progression, and finally secrete anti-inflammatory cytokines. Strategies to fight these macrophages include inhibition of the M2 phenotype as well as induction of M2-to-M1 reprogramming, as reviewed by Genard et al. [153]. Both polarization routes can be targeted with PARP inhibitors, but the documented involvement of poly-ADP-ribosylation in defining the M1 phenotype suggests the use of PARP traps (preferably not blocking catalytic activity) for the desired reprogramming. PARP inhibition was shown to dampen macrophage M1 polarization and to curb the release of cytokines that could negatively affect cancer growth or survival. On the other hand, inhibition of ADP-ribosylation was shown to increase the infiltration of proinflammatory macrophages characterized by pTBK1 and pIRF3 to pathological sites [82]. Furthermore, the enzyme facilitates the NFκB-dependent transcription of the *CCL2* cytokine, which acts as a chemoattractant to monocytes and TAMs recruited to the tumor to facilitate its growth [72,153]. Previous attempts to target CCL2 with the monoclonal antibody met with success in mouse models and humans. A similar effect was observed with CSF1, another chemoattractant that was captured with the antibody. *CSF1* requires PARP1 cleavage to be transcribed in macrophages; thus, inhibitors of NLRP3 might also be considered anticancer agents in this context. Since PARP1 facilitates the expression of IL4 upon T-responses in a model of atopic dermatitis, PARP inhibitors may indirectly interfere with IL4-induced M2 polarization [89]. Since PARP1 prevents the degradation of STAT6, it can possibly promote development of TAM [101,102]. Therefore, the inhibition or silencing of PARP1 in cancer cells may prevent or at least reduce the M2 polarization of adjacent macrophages. Importantly, there is one more piece of evidence for the contribution of PARP1 to PD-L1-dependent suppression of the immunological response to cancer cells [100]. The treatment with PARP inhibitors boosted PD-L1 expression in breast cancer cells, decreasing the number of tumor-infiltrating cytotoxic T cells in a xenograft model, whereas the combination of PARP inhibitors and anti-PD-L1 antibody reversed the immunosuppressant effect [154]. PARP1 may indirectly orchestrate the immune response evasion of cancer cells by affecting the expression of interferon genes in a DSB-dependent manner. It is well established that the expression of PD-L1 in cancer cells is directly controlled by IFN-dependent signaling [155]. Interestingly, PARP inhibitors triggered more considerable type I IFN responses in ERCC1-deficient lung cancer when compared to the wild type. PARP inhibitors niraparib, rucaparib, and talazoparib increased PD-L1 expression, which was additionally enhanced by IFN-γ treatment in lung and breast cancer cell lines. Notably, the synergistic effect was more robust in ERCC1-deficient lung and *BRCA1*-deficient breast cancer cells [156]. Moreover, PARP traps generated a substantial number of repairable DNA double-strand breaks due to defective homologous repair system (HR) in ERCC1-deficient cells, thereby leading to an accumulation of cytoplasmic DNA fragments, which, in turn, activated cGAS-STING signaling [156]. In mice bearing *Brca1*-deficient ovarian tumors, olaparib induced antitumor immunity involving both adaptive and innate immune responses through a STING-dependent pathway that was further enhanced by combination with PD-1 blockade [82]. Activation of the cGAS-STING pathway by PARP inhibitors boosts PD-L1 expression and, thus, cancer cells escape from the desired immune response. The effect of PARP inhibitors on PD-L1 expression highlights the need to combine these compounds with anti-PD-L1 treatment or PD-1 blockade to maximize the anticancer feedback from immune cells and improve the therapeutic outcome for cancer patients. Summing up, the decrease in PARP1 activity and ADP-ribosylation with inhibitors in cells such as macrophages, which directly impact cancer progression and may support current anticancer approaches and improve cancer treatment.

## 8. Conclusions

The collected reports indicate that PARP1 promotes the proinflammatory phenotype of macrophages. PARP inhibitors are capable of reducing inflammation. However, in the context of anticancer therapies the immunomodulatory role of the enzyme should also be considered in the light of PARP1 involvement in the endogenous cancer pathways and in the maintenance of genome integrity, which may lead to expression and release of factors proficient in directing macrophage polarization towards M2. The currently tested and approved PARP inhibitors can be used in other therapies once their action in particular disorders is confirmed. First, we need to overcome the negative side effects, which are, unfortunately, likely to occur due to the involvement of PARPs in numerous intracellular processes as well as in primary cells like monocytes, which are characterized by low PARP1 abundance. In any case, the discovery of molecular mechanisms that control PARP1 expression and the mode of PARP1 contribution to intracellular processes extends the list of possible compounds that can be used for targeted PARP1 interventions in a cell type- or pathway-specific manner.
